# Post-synaptic specialization of the neuromuscular junction: junctional folds formation, function, and disorders

**DOI:** 10.1186/s13578-022-00829-z

**Published:** 2022-06-19

**Authors:** Suqi Zou, Bing-Xing Pan

**Affiliations:** 1 Laboratory of Fear and Anxiety Disorders, Institute of Life Science, Nanchang University, Nanchang, 330031 Jiangxi P. R. China; 2 School of Life Sciences, Nanchang University, Nanchang, 330031 Jiangxi P. R. China

**Keywords:** Post-synaptic specialization, Neuromuscular junction, Junctional folds, Development, Formation mechanism, Function, Disease

## Abstract

Post-synaptic specialization is critical to the neurotransmitter release and action potential conduction. The neuromuscular junctions (NMJs) are the synapses between the motor neurons and muscle cells and have a more specialized post-synaptic membrane than synapses in the central nervous system (CNS). The sarcolemma within NMJ folded to form some invagination portions called junctional folds (JFs), and they have important roles in maintaining the post-synaptic membrane structure. The NMJ formation and the acetylcholine receptor (AChR) clustering signal pathway have been extensively studied and reviewed. Although it has been suggested that JFs are related to maintaining the safety factor of neurotransmitter release, the formation mechanism and function of JFs are still unclear. This review will focus on the JFs about evolution, formation, function, and disorders. Anticipate understanding of where they are coming from and where we will study in the future.

## Introduction

Chemical synapses enable cells communication via neurotransmitters release and induce the chemical signal pathway active in the receptor cells [1, 2]. The neuromuscular junction (NMJ) is a typical chemical synapse established between the lower motor neurons in the spinal cord and the skeletal muscle fibers. It comprises three essential elements: presynaptic motor nerve terminal; perisynaptic Schwann cells (SCs); and the specialization post-synaptic plasma membrane of the muscle fiber [3–6]. In the past, most of our understanding of synapse formation and receptor clustering comes from these researches at NMJ. For example, the conclusion that NMJ was a site of neurotransmission between separate cells claimed by Kühne was earlier than the neuron doctrine. And the ‘vesicular hypothesis’ proposed by Jose del Castillo and Katz explained how acetylcholine (Ach) is released by quantal from nerve terminals [5].

The significant difference between the NMJ and neuron-neuron synapses is located in the synaptic cleft and the post-synaptic membrane. The gap of NMJ in mammals is width from 50 to 100 nm, and the basal lamina (BL) is located in the middle of the cleft [7]. While the width of synapse in CNS is about 20–30 nm, and there is no BL existing [8]. Face the active zones (AZs) of the pre-synaptic axon terminal is the primary branch of JFs. At the shoulder areas of JFs crests, AChRs are concentrated at a high concentration of 10,000/um^2^ with 1000X highly than the extrasynaptic areas. At the center-most part of JFs crests, the adhesion molecule integrin α7β1 is anchored to laminin α4 of the BL and is crucial for the positioning of AZs of pre-synaptic axon terminal [9]. At the valleys of the branch of the JFs, voltage-gated sodium channels (VGSCs) are concentrated and are responsible for the action potential (AP) in the muscle cells. Nowadays, the signaling pathway of acetylcholine receptor (AChR) clustering on the NMJ has been studied well and reviewed [3, 6, 10, 11]. However, the function and formation mechanism of junctional folds (JFs) are unclear.

**Fig. 1:**
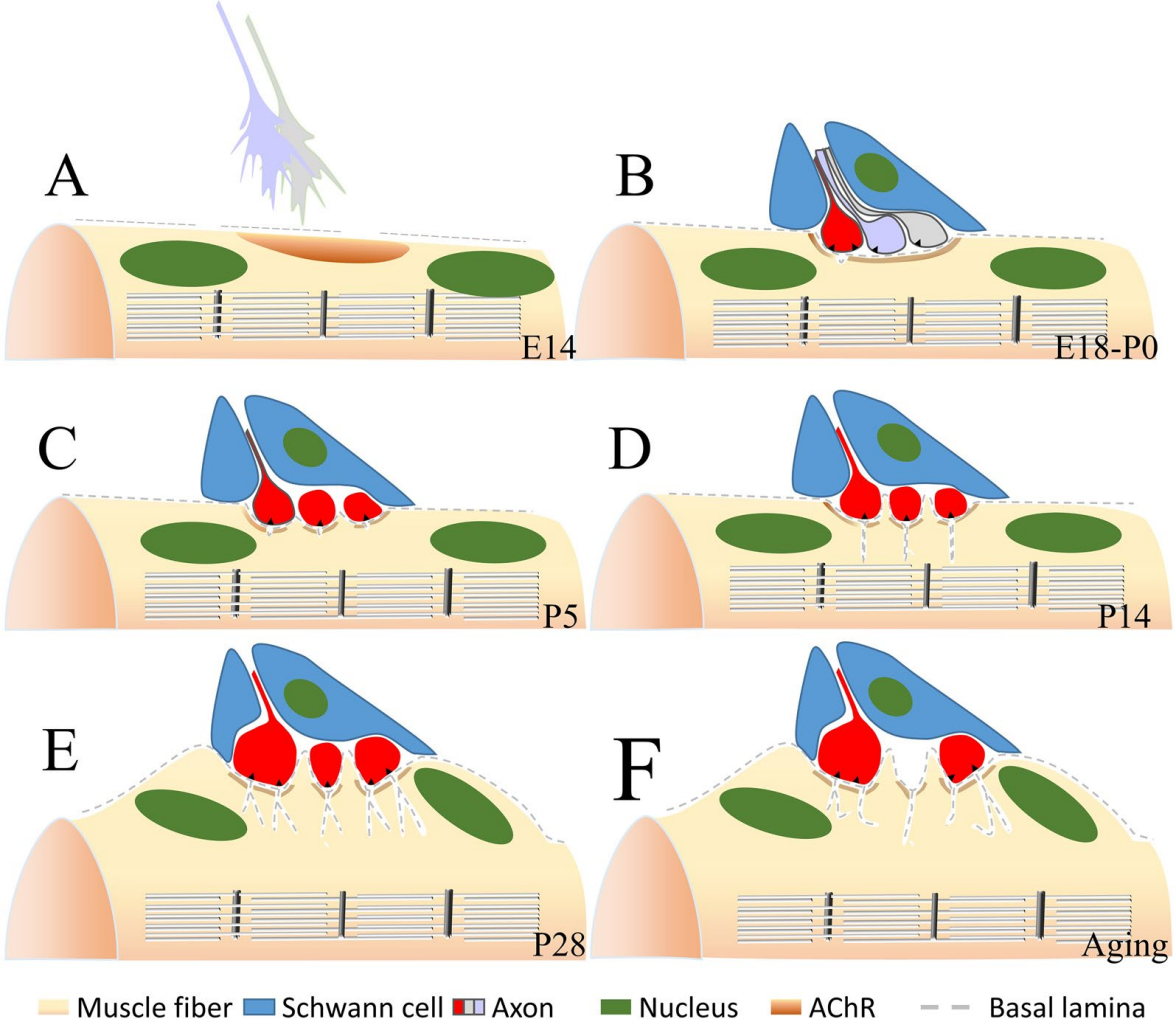
Morphological changes of the mouse JFs during development. ** A** Before E14, the axon terminal is not attached to the muscle fiber, and the AChRs are pre-clustering in the post membrane. BL materials already exist around the muscle fibers. **B** At the E18-P0 stage, multi-nerves target the muscle fiber and induce the post-membrane to formate a shallow gutter. Few pit-like incipient JFs are not aligned opposite to the AZs at this stage. **C** During the first 5 days, excessive axon terminals were eliminated, and pit-like JFs were the dominant structure. **D** Before the 14 days, JFs elongated into the subneural sarcoplasm. Sarcoplasmic without axonal terminals covered are upheaved and matured to the sarcoplasmic protrusions. **E** At the 28 days, almost all JFs matured to the slit-like shape, and with the secondly JFs. The entire post-synaptic apparatus have been raised above the surface of the muscle fiber and termed the “endplate”. **F** At the aging JFs, axon terminals are denervation gradually. Few second JFs degeneration could be found, but the primary JFs was always preserved. Unopposed JFs to the AZs are prevalent in aging

During the development of the mouse NMJ, the topography of JFs was under dramatic changes in the Z dimension in the first two weeks after birth [12]. At the E14 stage, BL is already present outside the sarcolemma before the motor nerve innervates. The sarcolemma is flatting without any depression, and the AChRs are pre-clustering in the post membrane (Fig. 1A). At birth, the NMJs are immature with simple round or oval depressions, and primitive synaptic troughs were smooth cup-like depressions 5–6 μm in diameter. Few pit-like or elongated oval invaginations and coated or uncoated caveolae were found beneath these depressions areas, indicating different section forms of the incipient JFs (Fig. 1B). At this stage, though the AZs were preceded the formation of JFs, they were not aligned with the JFs [13]. In the first 5 days, incipient JFs are still few and are primarily pit-like or elongated oval, but they are starting to invaginate into the sarcoplasmic with a depth less than 400 nm (Fig. 1C). Accompany excessive nerves were eliminated on the NMJ, low and narrow sarcoplasmic ridges occur in the cup-like depressions [14]. However, whether JFs are only present in those axons that will be preserved, or those axons that possess FJs will be more resistant to be pruned, requires further investigation in the future. At the P14, those low sarcoplasmic ridges were upheaved and matured into sarcoplasmic protrusions. Though the number of JFs has increased by about 18 folds, they were still predominantly pit-like and arranged in a row in a side-by-side fashion. The depth of JFs has also elonged and extended to a maximum of about 800 nm (Fig. 1D). At the P28, though few pits still coexisted in a small proportion, the JFs form was mostly slit-like shape [14], and all JFs are directly opposed to AZs of motor terminals (Fig. 1E). The entire post-synaptic apparatus was raised above the surface of the muscle fiber, which gave rise to the term “endplate” [3]. The length of specialized thickened membrane increased 1.5- to 1.8 fold, and the total number of endplate receptors increased over 30 folds [15].

## The evolution of junctional folds

### NMJ in the lower invertebrate animals

**Fig. 2:**
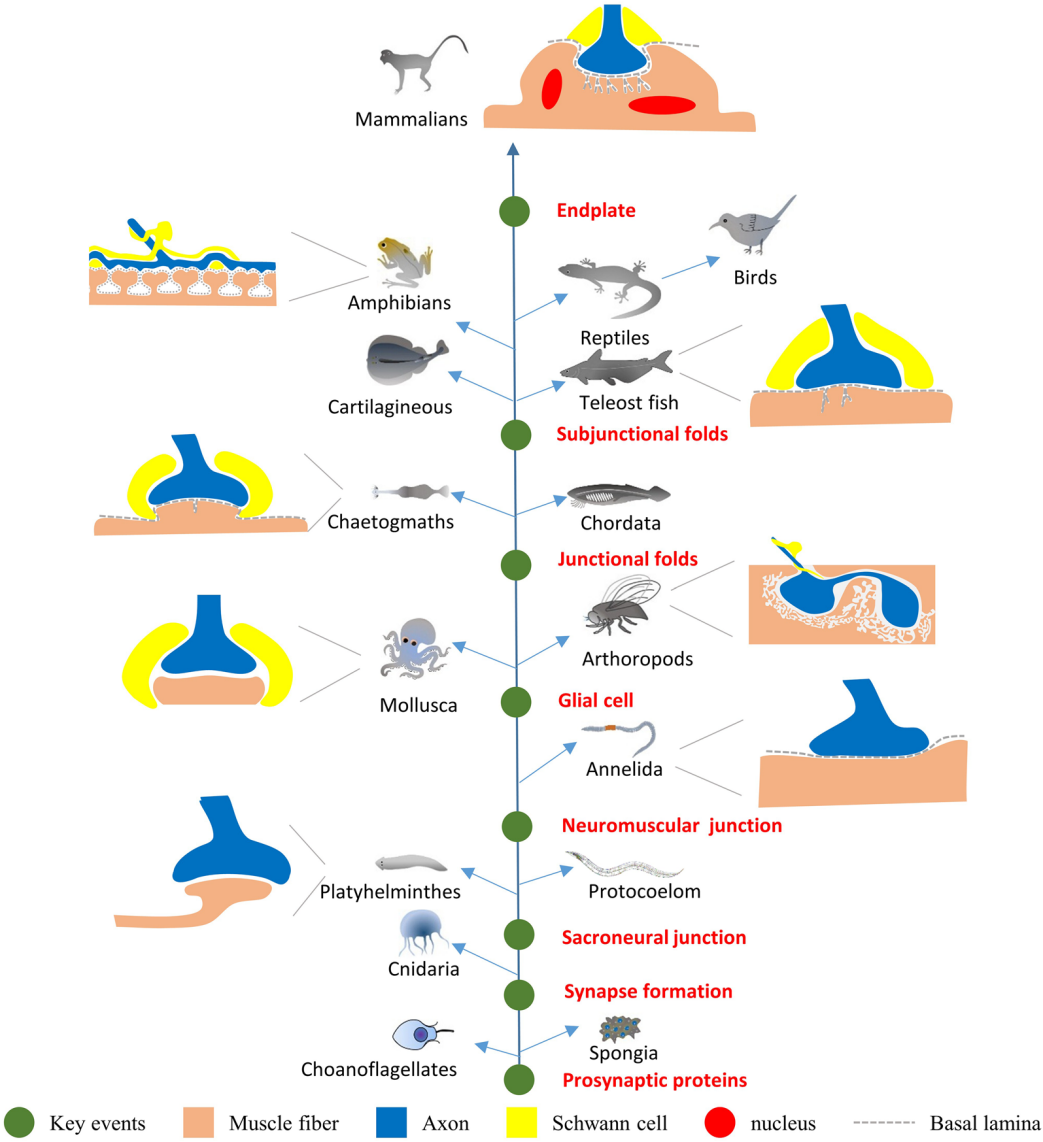
Phylogenetic tree of JFs evolution. Grey images represent representative species. The representative morphology of each animal JF is shown with a schematic diagram. The green dots represent critical events in the evolutionary history of JF. Protosynaptic proteins emerged in the *Choanoflagellates* and *Spongia*; the first NMJ emerged in the Annelida; glial cells participated in the NMJ structure in the *Mollusca* and *Arthoropods*; the first JFs of post-membrane depression emerged in the *Chaeogmaths* and *Chordata*; deeper and branched JFs were found in the *Cartilagineous* and *Teleost* fish; higher densities of JFs was found in *Amphibians*; the most complex of JFs structure existed in the mammalian animals

Though protosynaptic proteins were first to emerge in the *Fungi* and *Spongia* at 1220 to 766 million years ago (Mya) [16], the recognizable nervous system was first found in the *Ctenophore* and *Cnidarian* (Fig. 2). *Ctenophores* may be the first branch of the animal lineage, and little is known about the physiology of the ctenophore NMJ. Glutamate is the only well-validated neurotransmitter in the NMJ of this animal. In the *Cnidarian*, synapses contained naked axons and muscle fibers separated by a 13–25 nm gap containing intra-cleft filaments. Though vesicles in the axon terminals suggested that neuropeptides had evolved as neurotransmitters, it lacks the post-synaptic specializations of higher animals [17]. Unlike the vertebrates, synaptic transmission usually travels in bidirectional in the *Ctenophore* and *Cnidarian* [18].

In the planarian, the simplest animal that possesses a simple brain has the movement ability to prey. Muscle was under the skin and constituted the dermo-muscular sac [19], it extended sarcoplasmic processes to the nerves, which were named sarconeural junction. Their vesicles in the motor neuron axons are of two main types: small clear vesicles measuring 20 to 40 nm and larger osmiophilic granule-containing vesicles measuring 40 to 80 nm. In the protocoelom animals, such as *C. elegans* [20] and *Ascaris lumbricoides* [21], body muscles project processes to form the motor neural junction directly with the nerve ring. The nerve fiber contains clusters of vesicles and giant mitochondria in this synapse, and BL was found in the 50 nm synaptic cleft. Interestingly, although nematodes are more distantly related to vertebrates, they both use Ach as their neurotransmitter at the NMJ.

In the *Annelida*, the segmental nerves of *Lumbricus Terrestris* send axons into the body wall muscle layers and establish an 85–120 nm wide cleft that contains basement membrane material. As glial cells are not abundant in earthworm nerves, the smaller nerve branches and NMJs consequently are composed entirely of unmyelinated axons and the adjacent muscle cells [22].

In the *Mollusca (Aplysia)*, glial tissue often remains at the site of NMJ to cap the axon, and it enables axons by a single layer in the anterior aorta nerve. Besides, the synaptic cleft is widened, and a clear BL could be found. Type I is innervated by cholinergic axons to mediate inhibition, type II is innervated by serotonergic axons to mediate excitatory effects, and type III is innervated by glycinergic terminals for modulating excitatory inputs. In most of the three NMJs, caveolae under the postjunctional membrane were very common. Especially in the type III NMJ, strings of caveolae seem to be formed by successive invaginations of the sarcolemma, which seem like the JFs shape. However, as BL did not extend into these caveolae, that means it is not a really JFs [23]. In the *Octopus Vulgaris*, though the pre-synaptic and post-synaptic membranes at the junction area were thicked, no special folding of the subsynaptic membrane has been found [24].

In *Arthropods*, glial cells and BL were preserved in their NMJ. The main characteristic of *arthropods* NMJ is the axon terminal inserted into the muscle membrane and enlarged in a bots manner. In those *aquatic arthropods*, such as crayfish [25] and blue crab [26], terminal axons make synaptic contact with the muscle granular sarcoplasm. In some flying insects, such as *Sphinx ligustri* larvae [27], Wasp [28], Cicada [29] and Drosophila [30], the NMJ in the high-frequency muscle fibers shows post-synaptic membranes form laminated stacks and tubules. It occasionally continuity with the circumfibrillar endoplasmic reticulum and the plasma membrane, which is designated post-synaptic subsynaptic reticulum (SSR). What are the roles of SSR surrounding the pre-synaptic bouton is not clear now; it is believed to add to the electrical capacity of the NMJ or maybe is the site of the oscillating mechanism in high-frequency muscle fibers [29]. In most arthropods, glutamate is the excitatory neuromuscular transmitter, while GABA is the main inhibitory neuromuscular transmitter.

### JFs emerges in *Chaetognaths* and *Chordata*

*Chaetognaths* (arrow worms) are enigmatic zooplankton whose phylogenetic position remains elusive because they display dual morphological characters of protostomia [31] and deuterostomia [32]. In the skeletal muscles of the head, a type of muscle fibers that give rise to protrusions to cross the basement membrane and contact nerves. In these NMJs, the most striking feature is the development of JFs, which seemed to increase the post-junctional membrane’s surface. The synaptic cleft is about 50 nm in width and contains a dense material that is continuous with BL out of the NMJ, and the SCs partially ensheathe the nerve endings and face the muscle fiber [32]. However, the synaptic transmitters are currently unknown due to the lack of physiological data in *Chaetognaths* muscles or nerves.

In the *Chordata*, a typical muscular structure in the notochordal lamellae of *Amphioxus* has displayed the classic characteristics of periodic cross-striation. Histochemical results showed that acetylcholinesterase was found above this muscular structure, indicating that it was innervated by cholinergic motor neurons. Under the electron microscope, post-junctional membrane folds were found in these muscle fibers, suggesting the notochord of *Amphioxus* is a highly specialized hydroskelcton that supplied enough stiffer for its movement [33]. The above results showed that JFs appeared at least 500 Mya on earth.

### Matured JFs were found in vertebrate animals

ACh is the principal neurotransmitter at the vertebrate NMJ. In the electric organ of *Torpedo marmorata* [34], sparsely JFs show deep folds into the sarcolemma; however, complex subjunction folds have not been found. In the *Scyliorhinus canicula L.*, varying JFs are located in the muscle fiber membrane under the axon terminal. Especially in the fast-twitch white muscle fibers, the number of JFs was more numerous, and the depth of JFs was more than that of the red fiber. The multiple foldings of the subjunction membrane were found, which indicate that completely mature JFs were established [35]. Besides, numerous, deep, often branched post-synaptic membrane infoldings were also be found in adult *Lampetra fluviatils L* [36].

In some lower teleost fish, such as *Clupea sprattus L.*, *C. harengus L.*, *C. pilchardus L.*, and *Ameiurus nebulosus L.*, JFs are less complex, and there are no subjunctional folds [37]. In *Puffer fish* (*Tetraodon steindachneri*) [38], *Hippocampus hudsonius* [39], *Snake fish* [40], and *Carassius* [41], the post-synaptic membrane still without the deep infoldings characteristic of other twitch muscle fibers. While in other higher teleost, such as *conger eel* and *sturgeon Acipenser hueri*, complex JFs upon the myoseptal end of the white muscle fiber has been found [42]. Interestingly, the lungfish (*Lepidosiren puradoxu*) is the ancient ancestor of *amphibians* in the present world, and it has subjunction folds in white myotomal muscle fiber [43].

Interestingly, JF was not found in the larva stage of *Rana temporaria L., Ambystoma tigrinum*, and *Xenopus*. But more prominent in adults amphibians (*Triturus helveticus*) [37], ileofibularis muscle of the turtle (*Trionyx sinensis*) [44], and the transversus abdominis muscle in the garter snake [45]. In *alligator mississippiensis*, the pupillae muscle has only a few JFs [46]. In the avians and birds, post-synaptic foldings were shown in posterior latissimus dorsi, while not in the anterior latissimus dorsi muscle [47, 48].

The JFs structure of mammals is the most complex in the animal kingdom. Such as rodents [49], canine [50], primates [51], and even in the neonatal calves [52]. Additionally, in the pectoral muscles of bats (*Rhinolophus ferrumequinum Nippon*), mitochondria-moderate fibers have well-development JFs [53]. In the white fibers of bottle-nose dolphins, marked branching of JFs also extended deep into the sarcoplasm [54]. Interestingly, even in cultured muscle cells [55], self-organizing 3D organoids of humans [56, 57], and the space mammal animals [58], the structure of JFs is still be found. These results (Fig. 2) indicate that although JFs have species differences among mammals, they are not devolution in microgravity environments. However, what is the evolutionary function of JF? Broadly speaking, high densities of VGSC located in highly folded postsynaptic membranes can amplify the effects of neurotransmitter transmission. As an extreme example, the NMJ of humans performs a ‘nummular’ morphology and occupies the smallest ratio of the muscle fiber’s surface. But it has the largest folding index than fish, frogs, snakes, and mice, which ensures the effective neuromuscular transmission [59].

## The mechanism of JFs formation

The mechanism of NMJ formation is very unclear right now. Consider that JFs have emerged after BL and glial cells in evolution. All the factors from axon terminals, SCs, and BL can influence the formation of JFs.

### Signals from the axon terminal are vital for JFs initiated

Innervation by motor neurons is essential for the formation of JFs [60]. Denervation induces the JFs absent at E18.5 [61]. In the choline acetyltransferase (ChAT) null mutation, synaptic transmission is entirely and specifically blocked, the nerve terminals were extensively differentiated, and the AZs density was unaltered. However, synaptic maturation was delayed at subsequent stages, and the number of JFs was decreased by 60% at E18.5 [60]. Neuronal activation is necessary to promote Wnt secretion [62], and it is dependent on the Wnt ligand secretion mediator (Wls) [63]. Knockout the Wls in motoneurons, while not in the SC and muscle cells, induced NMJs to become unstable and JFs reduced. R-spondin 2 (Rspo2) is expressed highly in spinal motor neurons (SMNs) and can enhance Wnt receptors’ stabilization to activate Wnt/β-catenin signaling pathways. In Rspo2-knockout mice, the numbers of AZs and post-synaptic folds are decreased, resulting in the frequency of miniature endplate potentials (mEPP) being markedly reduced, and the mutant mice died shortly after birth due to respiratory distress. However, overexpressing Rspo2 in the SMNs did not increase the numbers of AZs and JFs. In contrast, overexpressed Rspo2 in the skeletal muscle, over-correcting all abnormal features of the post-synaptic region observed in Rspo2-/- mice [6]. Besides, as Munc is essential for the synaptic vesicle priming at AZs, the fusion-competent synaptic vesicles were decreased in Munc13-1/2-DKO mice. Interestingly, the JFs invaginations are no deep as the normal mice, and it is small and shallow at the post-synaptic membranes of NMJs [64].

However, axons innervated are unnecessary for JFs maintenance. In the diaphragm of rats, ‘empty’ folds are still found at 4–6 months after the phrenic nerve section, indicating that JFs are very stable once the muscle fibers are denervated [65]. Especial the deep primary synaptic grooves and the secondary synaptic clefts were both present 12 weeks after denervated [66]. Furthermore, after denervation of the rat soleus muscle at P2, those denervated muscle fibers could still be programmed to form folds and showed marked JFs at P5. At P15, the endplates stopped JFs formation, and the post membrane was completely smooth [67].

### SC indirectly affects the formation of JFs

The myelin sheath of Schwann cells is very beneficial for action potential conduction and neurotransmitter release. In the erbb2-deficient mice, SCs were absent in the phrenic nerve, and the NMJs were dysfunctional, resulting in the mutant mice dying at birth. Immunological examination shows that the post-synaptic membrane of the mutants lacked JFs at E18.5, while the JFs in control mice were significant [68]. In the erbb2/4 double knockout mice, nerve terminal endings appeared normal while the JFs were scarce or even lacking, and the number of fold openings per synaptic contact length was decreased in mutant mice [69]. In the conditional knockout model, ablated SCs at E15.5 did not influence the vesicles and AZs in the axon terminal, the synaptic width, and the AChR cluster’s density. But the amplitude of mEPPs and JFs depth were reduced [70]. However, acutely killing the perisynaptic SCs in adult mice with anti-disialoside antibodies has no significant deleterious influence on JFs maintains [71]. Thus results show that SCs may indirectly influence the formation of JFs through the synaptic transmission.

### BL is essential to the forming and maintaining of JFs

In those muscle fiber injured animals, regeneration muscle cells form new JFs in the original location under the guidance of surviving laminal skeletal, even though the axon was absent [10, 72–74]. This indicates that BL has vital roles in the JFs maintenance [75].

In the basal membrane of the skeletal cell, laminin is a crucial component and contains α2, α4, α5, β1, β2, and γ1 subunits [76]. Laminin α2 is located throughout the skeletal muscle surface, and mutant mice display motor neuron terminals that withdraw from the endplate and small JFs. However, the number of AZs and the proper location as opposed to the JFs were normally [77]. Laminin α4 is located in the BL of NMJ, where AZs are absent and do not localize at the gutter of JFs. Laminin α4 knockout mice perform normal JFs and AZs, while the locational of AZs is misaligned [78]. Laminin α5 is concentrated at the synaptic cleft shortly after birth. Specific knockout laminin α5 in skeletal cell display arrested post-synaptic maturation of NMJs to the early postnatal stage. Double knockout mice of laminin α4/α5, NMJ innervated was not found once the competitor axons withdrew from the AChR territory, and these mice were smaller and weaker and survived only three months of age [79]. Laminin β1 and Laminin β2 have opposite locations, and the former is found throughout the extrasynaptic BL while the latter is enriched explicitly at the BL of NMJs [80]. In the Lamβ2-/- mice, the NMJ area or morphology was normal, while the axon terminal has fewer AZs and displays some drawbacks, the SCs extend processes into the synaptic cleft, and the JFs are absent. Rescue β2 expression in muscle restored NMJ architecture and improved the weight and lifespan [81].

The major secreted heparan sulfate proteoglycans (HSPGs) in the BL are Agrin and Perlecan [82, 83]. Loss of agrin is lethal to the mice as the acetylcholine receptor could not cluster by the musk pathway, and the JFs was disappeared in the knockout animals [84]. Perlecan is also riched within the basement membrane surrounding skeletal muscle fibers, and it is the acceptor for collagen-tailed AChE that binds to the synaptic BL. In the perlecan-null mice, the AChR cluster and prominent markers of the post-synaptic JFs were normal, while the AChE clustering was utterly absent. Thus perlecan seems not to affect the organization and localization of the post-synaptic membrane [85]. Similar results were also found in the AChE−/− mouse, that JFs were well-formed [86]. However, though AChE was absent in the Colq−/− mutant mice, some subsynaptic cytoplasm appeared necrotic, the JFs were utterly reduced, and most mutants died before they reached maturity [87].

Matrix metalloproteinases are the critical regulators of the extracellular matrix. Secretion of pro-MMP3 is produced by the skeletal muscle and the SC and is concentrated at NMJ extracellular matrix through a hemopexin domain. In the MMP3 null mutant mice, the agrin immunofluorescence was significantly increased at NMJ, and the AChR aggregates were increased. Electron microscopy revealed no difference in the pre-synaptic nerve terminal, while the number and length of JFs were dramatically increased. As in atypical locations, the JFs extent is adjacent to the subsynaptic nuclei of the muscle cell [88]. Notably, tissue inhibitors of matrix metalloproteinases (TIMPs) are the special inhibitor that binds to the regulated the active of MMP3. The synaptic activity regulates TIMP release, that synaptic activity decrease will increase the TIMP release [89].

Fgf18 is expressed in the motor neuron and is also localized at the BL of NMJs. In the Fgf18−/− mice, synaptophysin-positive areas decreased to one-third of the wild-type level. In the post-synaptic membrane, simplified endplates with litter AChR positive areas and significantly fewer JFs [90]. Collagen IV (α2, α3, α6 chains) and collagen XIII are concentrated at mouse NMJs. They are required for synaptic maintenance [91], but not crucial to the JFs formation. Although the number and depth of JFs were not analyzed in collagen XIII mutant mice, some “naked” post-synaptic without axon terminal innervated, and others post-synaptic covered by SC processes were found [92]. Collagen XIII overexpression mice did not show any defect in the amplitude and frequency of mEPP, and the JFs number seems no different [93].

In addition, molecules involved in AChR clustering also affect the formation of JFs. In the ε AChR mutant mice, the AChR could not transform to the adult type, and the synaptic could not maintain the highly organized structure of the neuromuscular endplates. The post-synaptic membrane was flattened and without the characteristic JFs at P60, which induced those mutant mice always to die prematurely [94]. Conditional ablated the Lrp4 gene in the adult mice induced the AChR clusters fragmentation and also caused the JFs ablation [95].

### Invagination mechanism of JFs formation.

**Fig. 3:**
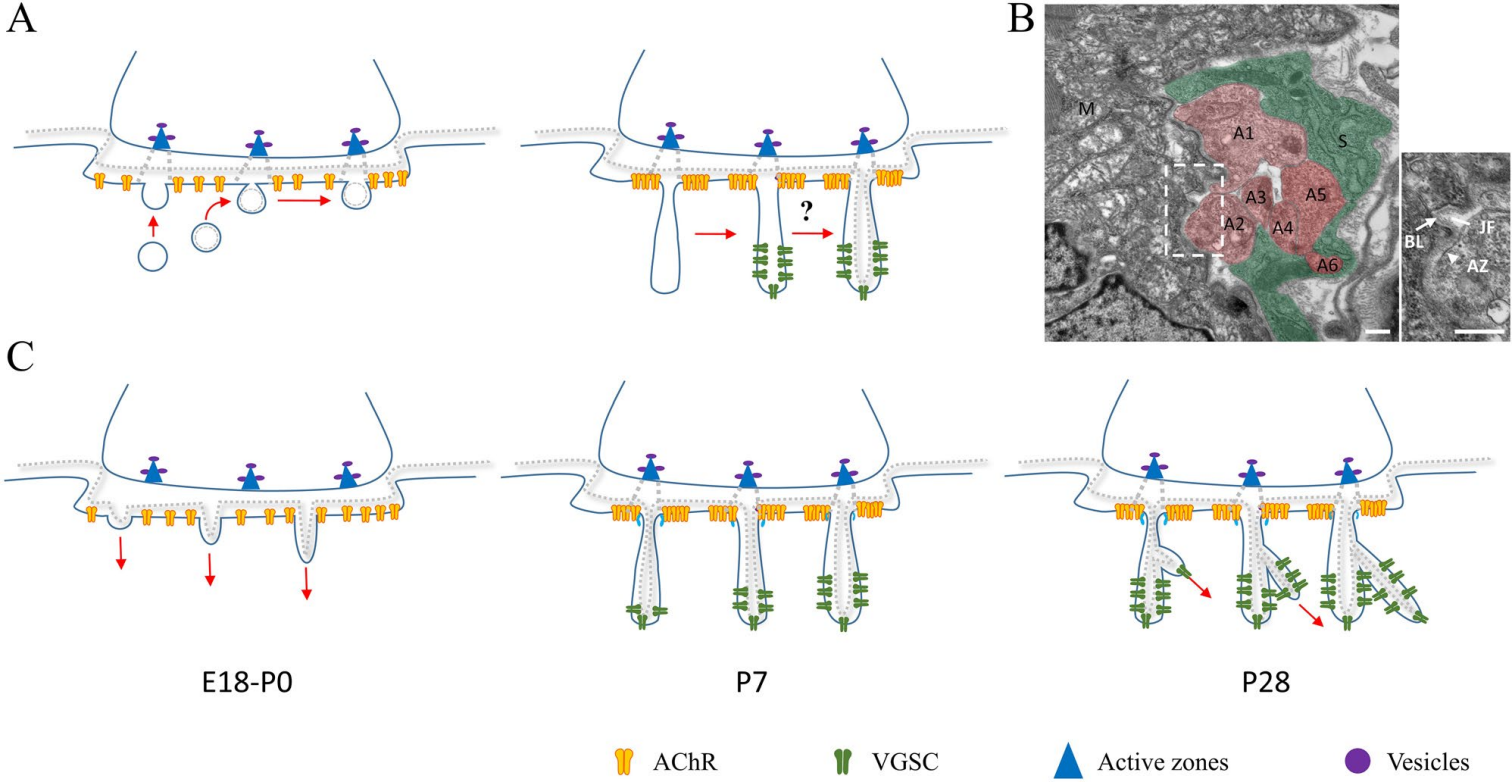
Hypothesized mechanisms for JFs formation. **A** Exocytosis model modified from the previous hypothesis. E18.5-P0, beneath the depression areas, numerous caveolae with coated or uncoated were found. As these caveolae fused to the post-membrane opposed sites to the active zone, incipient JFs emerged on the stage. Accompanying more new membranes were inserted at P7-P28, the post-synaptic membrane investigated deeper into the cytoplasm and formed the maturely JFs [10]. However, this hypothesis can not explain how the BL are inserted into the JFs (question mark), especially why the secondly JFs formation. **B** The diaphragm of mice at P1 was observed by transmission electron microscope. Although Schwann cells (green) encapsulate multiple axons (red) in a single NMJ, only a single JF was observed beneath the NMJ. The incipient JF showed apparent invagination features, and the BL (arrow) within the JF maintained continuity with the outside. The inset figure is an enlarged view. Note that the active zone (arrowhead) is not opposed to the JF. **C** Invagination model was proposed by this reviewer. At E18-P0, the AZs were preceded by the formation of JFs, and they were not aligned with the JFs [13]. Initiated single from the axon terminal induced cytoskeleton pulled the sarcolemma investigated into the cytoplasm. In the first week after birth, the incipient JFs aligned the active zone to the opposed site with the interaction with BL, and the VGSC integrated into the valley of JFs. In the first 4 weeks after birth, the second JFs formed, which keep this mature structure to the adult stage. S: Schwann cell, M: Muscle fiber, A: Axon. The scale bar in B is 500 nm

What is the mechanism of JFs formation is very little known. A widespread explanation is that the newly formed membrane during muscle fiber growth is inserted into the post-synaptic membrane opposite the AZ, which then induces intercalary expansion of the post-synaptic membrane and finally induces the post-synaptic membrane to fold into the cytoplasm (Fig. 3A) [10]. The main feature of this theory is that JF must be formed on the opposite side of AZ. Typically, however, JF is not the first development on the site opposite AZ (Fig. 3B) [14]. And some caveolae composed of BL under the post membrane may be the cross-section of incipient JFs [96]. This mechanical explanation could not be enough to explain the formation of JFs. Especially, this hypothesis could not explain how the subjunctional folds are formed. Contract to this exocytosis hypothesis, an invagination mechanism (Fig. 3C) will be more reasonable. As noticed that BL in most omega-shaped coated caveolae under the cell surface is continued with the extra BL (Fig. 3B), which means this shallow depression was under invaginating into the cytoplasm [96].

The key question to understanding the invagination mechanism is what mechanism pulls the post-membrane inside. In evolution, the first sodium-selective channels may have appeared in extant cnidarians at the 700 Mya [97], and it evolves ankyrin binding in *Amphioxus* about 520 Mya [98]. Interestingly, JFs first emerge in *Chaetognaths* and *Amphioxus* at a similar period (about 500 Mya). JFs formation may be sharing the common mechanism that exists throughout the CNS for clustering VGSCs at a high density [99–102]. It was reported that β-spectrin binding to ankyrin maintains the high local density of VGSCs in the Ranvier node and the axon hillocks of CNS. In Drosophila, TEM results show that the density of SSR was significantly decreased in β-Spectrin mutant [103]. Ablating a single exon in the spectrin repeat region of the *Kalrn* gene also decreased the JFs fold density in KalSR^KO/KO^ mice [104]. Recently, mice with ankyrins-deficient muscles only lost NMJ Nav1.4 performed significantly less voluntary movement and fatigued more quickly [105]. Besides, the postsynaptic intermediate filament network under ankyrin proteins is vital to the structural integrity of NMJs. Conditional knockout *plectin* in muscle cells shows severe muscle weakness and scattered few JFs (mostly curved and disoriented) [106]. In addition, AnkG and AnkR appeared to be present exclusively in the troughs of JFs [107, 108]. Whether ankyrins coupled with the VSGC to Spectrin/Plectin are involved in initiating JFs invagination needs more research in the future.

## The function of JFs

As described above, two facts need to be burned into our minds. First, JFs widely exist in different species of fish, amphibians, reptiles, and mammals, which means it is a choice of the convergent evolution. Second, there are so many species on the earth without JFs still thrive well, and even mutant mice that lack the JFs only produce weakness while not vital immediately after birth [81]. Though some morphology hints indicated that JFs contained BL deeply insect into the skeletal cells could retain the spatial location nerve terminal atop the muscle cell no matter the muscle cell is twitching or not [89]. However, what is the function of JFs? There is no clear conclusion yet.

### The safety factor of neuromuscular transmission

To understand the role of JFs, we should revisit the progress of how the nerve AP induced the skeletal contraction in more detail. In the beginning, AP arriving at the pre-synaptic nerve terminal usually activated approximately 5% of AZs and released approximately 60 ~ 80 vesicles from the docked vesicles pool (approximately 1200 ~ 1600 docked vesicles in the adult mouse NMJs) [109]. As the pulse of ACh crosses the cleft at a very high concentration (about 10mM), it will arrive at the post-junctional critical area within 15 usec by free diffusion and not degradation by AChE on its path [110]. Once the ACh binds to the ligate gate ion channel of AChRs on the sarcolemma, it induces the channel pores to open, and non-specific conducts for Na^+^ accompanied by some Ca^2+^ influx and K^+^ efflux [111]. Generally, a single quantal content activated about ~ 0.3 um^2^ ‘critical area’ of AChR in the post-junctional area generating a minEPPs about 0.5 ~ 1 mV in amplitude. In mouse and rat NMJs, the quantal content is about 40–100, while the humans usually are about 20, so the activation of endplate areas occurs at < 10% of the total NMJ areas. The number of minEPPs sum to generate an EPP of about 20–35 mV in rat and mouse NMJ, which is larger than the minimally needed to trigger the sodium channel open in the depths of JFs (about 10–12mv). Once the mount of VSGCs was opened in the bottom JFs and the post membrane depolarization reached the threshold, an AP was initiated successfully. AP travels bilaterally on the muscle fiber membrane and then invades the T-tubular system to trigger the muscle fiber contraction [110]. The efficiency of neuromuscular transmission largely depends on the safety factor (SF), defined by the EPP divided by the minimum amplitude of the initiated threshold minus the resting potential. SF is varied considerably among mammal species [112, 113]. In human NMJs, the SF is about 2, while the mouse and rat NMJs range from 1.8 to 6. This SF existing could ensure that the EPP amplitudes remain suprathreshold after a series of nerve activities; even the quantal content of ACh tends to decrease during intense muscle contract. In other words, it means that SF makes muscles less prone to fatigue during continuous exercise [111]. The NMJs of extraocular muscles, which have less prominent JFs and a reduced SF, make them more susceptible to developing myasthenic weakness than fast-twitch skeletal muscle fibers [114].

### A “saltatory conduction” model of AP spreading during the JFs

**Fig. 4:**
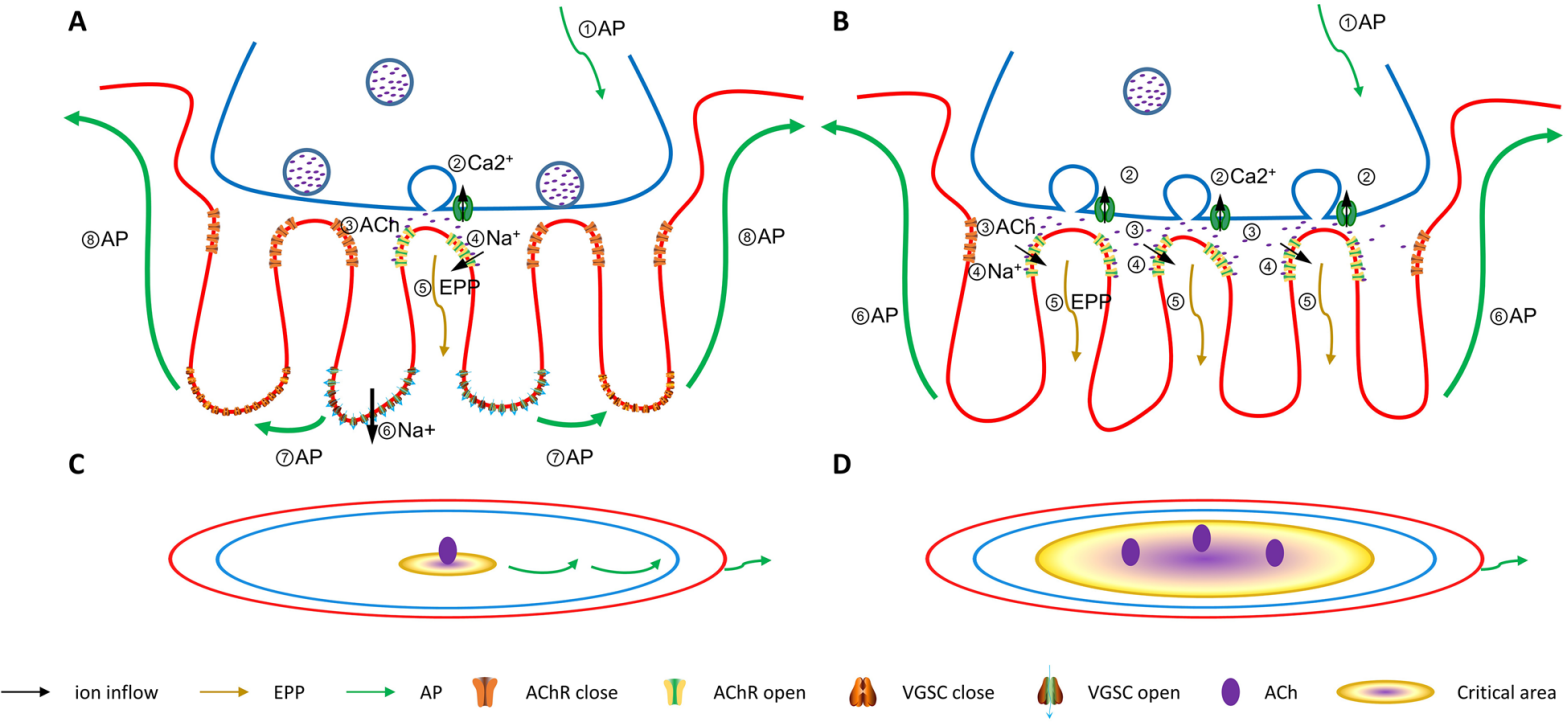
Diagram of AP conduction within the NMJ. **A** In the normal NMJ, (1) a nerve AP arrives at the nerve terminal and produces rapid depolarization, (2) VGCCs opening and Ca^2+^ entry, (3) the transmission vesicles fusion to the pre-membrane and release ACh into the cleft, (4) ACh bind to their post-synaptic receptors and generate a localized EPP at the crest of JFs, (5) EPP arrive the valleys of JFs and produces rapid depolarization, (6) VGSCs opening and generate of a muscle AP, (7) VGSCs on the neighbor JFs induced opening by voltage gating, resulting saltatory conduction happening, (8) Orthogonally aligned JFs propagate the AP along the long axis and drive the muscle fiber contraction. **B**. In VGSCs deficient JFs, muscle AP is not initiated by the Na^+^ inflow of VGSCs. To spread the AP throughout the whole NMJ region, it needs to activate more VGCCs and release more ACh vesicle quanta. Figures **C** and **D** are the plane views of **A** and **B**, respectively

As described above, a single nerve impulse active a small critical area of the total NMJ (less than 10%). However, the details of AP spreading during the JFs were not mentioned before [102]. To simplify our model, we assume that a single AP induced by several ACh vesicles quanta was formed on the crest of one JFs (Fig. 4A). AP arrives at the axon terminal in the normal NMJ, inducing the VGCCs open and ACh to be released into the synaptic cleft. Then, AChRs of “critical areas” are activated, and EPP was initiated attributed to net sodium ion inflow. Then, the EPP is conveyed to the valleys of JFs and induced AP formation by sodium ion inflow, which will depolarize the post-membrane of nearby JFs. Then, the VGSCs of neighborhood JFs were initiated to new AP by a positive feedback pathway. During the AP transmission between the JFs, the cleft between the JFs and axon terminal should be insulation as AChR are ligand gating while not voltage gating channel [102, 115]. If VGSCs are deficient on the JFs (Fig. 4B), the AP of the nerve terminal induced more VGCCs open and more ACh vesicles quanta release, active more extensive areas of “critical areas” to produce a higher EPP for inducing enough muscle contraction. Stimulation of continuous neural signals causes VGCCs overload in the pre-synaptic membrane, resulting in the muscle being unresponsive to AP, and more accessible to fatigue. These explained that though the loss of JFs did not induce animal death immediately, muscle fatigue might be the common characteristic of JFs insufficient [105]. However, the specific function of JFs needs more detailed research to do in the future.

### The delicate structure of JFs ensures the possibility of saltatory conduction

During the progress of the AP spread within NMJ, JFs may have effects in several ways. Firstly, as highly aggregated AChRs are located at the shoulder of JFs, the constant quantal of AChR release will induce more AChR channels to open and increase the EPP’s size. Moreover, the density of VGSCs is only located at the trough of the secondary post-synaptic folds, which will increase the membrane excitability and reduces the AP threshold. Both of them ultimately result in increased SF magnitude [59, 116]. Secondly, the narrow cytoplasmatic space of JFs forms a high resistance pathway for the EPP induced current, and more VGSCs concentrated in the trough of JFs increased the maximum sodium channels conductances, both resulting in the current being easier to convey toward the depths of the secondary synaptic folds [99]. Thirdly, in the cleft between the crest of JF and the pre-synaptic membrane, integrins on the post-synaptic sarcolemma could bridge a physical connection to the axon terminal through the BL. This physical connection will be analogous to the membrane diffusion barrier that exists (paranodal axoglial junction) in the node of Ranvier, that borders the AChR and VGSCs laterally diffusion [117]. Last but most important, as the AChR channel is a ligand gating channel but not voltage-gated, and a large amount of BL exists in the cleft space on the top of JFs, this space will be an electrically insulated area for the AP transmission [115].

Benefit from this delicate structure, slight structural variation in synapses could not induce observable current transfer defect because the high density of post-synaptic Nav1.4 in the JFs has compensation roles [105]. In the Lambert-Eaton myasthenic syndrome, the P/Q-type VGCC of the motor neuron terminal was attacked by autoantibodies, and the ACh released was reduced. Ultrastructure results show that JFs were deeper than the normal group, which means more VGSCs will be concentrated in the deep of JFs [118]. However, in the homozygous SCN4A mutation (p.R1454W) patient, the probability of muscle AP initiation and propagation would be reduced as all sodium channels are mutated, which leads to fatigable muscle weakness [115, 119]. Recently, perfect research shows that only loss of Nav1.4 in the NMJ induces the mice to perform voluntary movement reduce and fatigued more quickly, while no defects of NMJ morphology and muscle strength [105]. We risked an interesting guess that JFs may have disappeared in this animal model.

## JFs disorders in NMJ related diseases

Defects in JFs are a common phenotype of many diseases that affect the NMJ, such as Myasthenia gravis (MG), Muscular dystrophy (MD), Amyotrophic lateral sclerosis (ALS), and aging [9, 120].

MG is an autoimmune syndrome in which neuromuscular transmission fails as NMJ maintenance signals are attacked by autoantibodies [121]. In the early onset stage of this MG, autoimmune antibodies in situ masking the membrane receptors induced the AChRs internalization and depression at the crest [122]. In the moderate and severe group, followed by systematic destruction, the receptor-containing crests of the JFs were destructed, and the JFs were degenerated, which resulted in the post-synaptic membrane was simplification [123]. In LG2 agrin mutation-induced MG, biopsy results from the right deltoid muscle showed that the JFs were simplified, and the diameter of the primary and secondary synaptic clefts was increased [124]. In Lrp4 antibody positive Myasthenia, most endplates had poor development, and JFs degenerated in some appeared denuded of nerve terminals [125]. In Musk inject rat, the sarcolemma of these NMJs is markedly simplified with sparse synaptic folds, and the number of secondary endplate folds per length of the primary cleft was significantly reduced [126]. SEM results from MuSK-injected mice also showed the subneural apparatus lost the labyrinthine structures, and the number of slit-like JFs was markedly decreased [127, 128]. In the Rapsyn Mutations induced congenital myasthenic syndrome of mice and patients, the JFs structure had poor development with few numbers and more short [129, 130]. Especially the secondary clefts did not establish continuity with the primary folds [131]. [132].

MD is a group of numerous genetic diseases characterized by progressive weakness and skeletal muscle degeneration. Dystrophin-associated protein complex (DAPC) is crucial for the integrity of sarcomeres and prevents its fragile from contraction-induced injuries [133]. Dystrophin deficiency causes symptoms similar to Duchenne muscular dystrophy (DMD) [134]. This dystrophin mutant mouse has a similar life span to wild-type mice, and the pre-synaptic component of nerve terminals and vesicle density had no significant differences. But the number and depth of JFs were reduced to 50% of normal [135]. The length of subjunctional folds was decreased significantly in the fast and slow fiber of MDX mice [136]. Utrophin is homologous of dystrophin and concentrates in the NMJ areas, and is thought to play roles in promoting post-synaptic membrane invagination. *Utrn-/-* mutants induced the numbers of JFs significantly reduced, which is similar to Duchenne and Becker muscular dystrophy [137]. Especially in the severely muscular dystrophy model of MDX:urtn-/- double mice, the numbers of JFs are notably absent [138, 139]. Syntrophins associate directly with the dystrophin protein family (utrophin, dystrophin, and dystrobrevin). Though the number of postjunctional folds was not reduced in the α-Syn-/- mice, their JFs displayed minor organization and had fewer openings to the synaptic cleft [108]. Biglycan is an extracellular matrix protein that regulates the dystrophin/utrophin protein complex localization. Several defects of NMJ are observed after P35, including increased segmentation of NMJ, the presence of perijunctional folds, and focal misalignment of AChRs and AChE [140]. In addition, as a laminin receptor on the posterior membrane, integrin α7 knockout mice lose post-synaptic JF and exhibit muscular dystrophic myopathy [76].

ALS is characterized by an adult-onset progressive of motor neurons death, and the patients often have atrophy and death within five years from diagnosis [141]. The JFs length of the outer compartment of TA was shorter in the SOD1 mutant ALS mice [142], and the number of JFs was often missed in the gastrocnemius muscle of Fus-/- mice at E18.5 [143]. Besides, in a mouse model of spinal muscular atrophy of SMAD 7 mutant mice, the JFs are almost wholly lacking [144]. Interestingly, junctional folds length decreased are more susceptible in fast muscles than the slow muscles in the early stages of ALS mice and patients [142]. Consider that JFs in fast fiber were deeper and more numerous than those in slow fiber, while the mitochondria in fast fiber were less than in slow fiber. And noticed that NMJ alterations were the early onset of the clinical symptoms [145], a dying-back hypothesis in ALS pathogenesis was proposed that NMJ dismantling plays a crucial role in starting ALS [120, 141]. Whether JFs function plays an important role in the onset of ALS requires more research in the future.

Although the elderly show some common features of ALS patients, such as nerve terminal denervation and muscle mass and strength reduction in gradually [141, 146]. The complexity of JFs in aging rodents and human NMJs had increased. Comparing the invasion of SC processes into the synaptic cleft, unopposed junctional folds to the AZs were prevalent [146]. JFs degeneration was few observed in the aging mice and patients [51, 147, 148].

Whether defects of JFs play a vital role in these diseases has not been investigated. However, two key questions need to be explored in each condition. Whether the JF phenotype is earlier than the NMJ phenotype? And does affecting the structural stability of JF produce these disease-related phenotypes? However, as the manipulation methods to regulate the JFs were very poverty, the exact role of JFs during these diseases is not well understood [149].

## Conclusions

The JFs were first observed in the Chaetognaths (arrow worms) at 500 Mya, and it was extinguished at a wide range of species, indicating that JFs were formed by convergent evolution. Though some cetaceans and bats live in the microgravity environment, their JFs structure did not devolve, suggesting that JFs were vital to animal survival on the earth. Considering that the past hypothesis of vesicle release cannot explain the development of JF, we propose an invagination mechanism in which the JFs were pulled into the cytoplasm by intrinsic or extrinsic mechanisms. Also, we give a concept that the VGSCs concentrated at the bottom of JFs participate in the spread of AP from the critical areas to the whole NMJ areas by a positive feedback way like the saltatory conduction in the Ranvier node, which ensures that the SF magnitude remains constant under physiological conditions. Understanding the formation mechanism of JFs will provide a clear direction for our future research.

## Data Availability

Not applicable.

## Abbreviations

ACh: Acetylcholine
AChE: Acetylcholinesterase
AChR: Acetylcholine receptor
ALS: Amyotrophic lateral sclerosis
AP: Action potential
AZs: Active zones
BL: Basal lamina.
ChAT: Choline acetyltransferase
CNS: Central nervous system
DAPC: Dystrophin-associated protein complex
DMD: Duchenne muscular dystrophy
EPP: Endplate potentials
HSPGs: Heparan sulfate proteoglycans
JFs: Junctional folds
Lgr5: Leucine-rich-repeat-containing G-protein coupled receptors
MD: Muscular dystrophy
mEPP: Miniature endplate potentials
MG: Myasthenia gravis
Mya: Million years ago
NCAM: Neural cell adhesion molecule
NMJs: Neuromuscular junctions
Rspo2: R-spondin 2
SEM: Scanning electron microscopy
SF: Safety factor
SC: Schwann cell
SMNs: Spinal motor neurons
TEM: Transmission electron microscope
TIMPs: Tissue inhibitors of matrix metalloproteinases
VGCCs: Voltage-gated calcium channels
VGSCs: Voltage-gated sodium channels
Wls: Wnt ligand secretion mediator
